# Imaging immunity in patients with cancer using positron emission tomography

**DOI:** 10.1038/s41698-022-00263-x

**Published:** 2022-04-07

**Authors:** Fiona Hegi-Johnson, Stacey Rudd, Rodney J. Hicks, Dirk De Ruysscher, Joseph A. Trapani, Thomas John, Paul Donnelly, Benjamin Blyth, Gerard Hanna, Sarah Everitt, Peter Roselt, Michael P. MacManus

**Affiliations:** 1 Department of Radiation Oncology, Peter MacCallum Cancer Centre, Melbourne, VIC Australia; 2 The Sir Peter MacCallum Department of Oncology, University of Melbourne, Melbourne, VIC Australia; 3 Department of Chemistry, University of Melbourne, Melbourne, VIC Australia; 4 Department of Cancer Imaging, Peter MacCallum Cancer Centre, Melbourne, VIC Australia; 5 Department of Radiation Oncology (Maastro), GROW School for Oncology, Maastricht University Medical Center, Maastricht, The Netherlands; 6 Cancer Immunology Program, Peter MacCallum Cancer Centre, Melbourne, VIC Australia; 7 Department of Medical Oncology, Peter MacCallum Cancer Centre, Melbourne, VIC Australia

**Keywords:** Cancer imaging, Tumour immunology

## Abstract

Immune checkpoint inhibitors and related molecules can achieve tumour regression, and even prolonged survival, for a subset of cancer patients with an otherwise dire prognosis. However, it remains unclear why some patients respond to immunotherapy and others do not. PET imaging has the potential to characterise the spatial and temporal heterogeneity of both immunotherapy target molecules and the tumor immune microenvironment, suggesting a tantalising vision of personally-adapted immunomodulatory treatment regimens. Personalised combinations of immunotherapy with local therapies and other systemic therapies, would be informed by immune imaging and subsequently modified in accordance with therapeutically induced immune environmental changes. An ideal PET imaging biomarker would facilitate the choice of initial therapy and would permit sequential imaging in time-frames that could provide actionable information to guide subsequent therapy. Such imaging should provide either prognostic or predictive measures of responsiveness relevant to key immunotherapy types but, most importantly, guide key decisions on initiation, continuation, change or cessation of treatment to reduce the cost and morbidity of treatment while enhancing survival outcomes. We survey the current literature, focusing on clinically relevant immune checkpoint immunotherapies, for which novel PET tracers are being developed, and discuss what steps are needed to make this vision a reality.

## Introduction

In an era of rapidly-evolving cancer management paradigms, made possible by our growing understanding of the fundamental drivers of cancer biology, the importance of the tumour microenvironment (TME), especially the contributions of the innate and adaptive immune systems, to cancer initiation, progression and metastasis, has been recognized. As our understanding of the role of the immune system in cancer has grown, highly effective new immunotherapeutic strategies have developed. In parallel with these scientific and therapeutic developments, our capacity to image components of the immune system with novel PET tracers has grown into a very promising area of research and development with the capacity to inform therapeutic decision making.

In this review we focus on the use of immune PET imaging to characterise clinically-relevant molecular targets, including those expressed on tumour cells and in the TME, with an emphasis on the therapeutic use of immune checkpoint inhibitor (ICI) immunotherapies, the most widely used of the new immunomodulatory therapeutics^1^. These agents recruit the patient’s own immune system to cause regression of cancer and have yielded meaningful, sometimes dramatic, objective clinical responses in diseases with previously bleak prognoses. Many patients have achieved prolonged disease-free survival and a proportion of these may even have been cured. ICIs, such as durvalumab and atezolizumab, targeting programmed cell death ligand-1 (PD-L1), and nivolumab and pembrolizumab, targeting programmed cell death protein-1 (PD-1), are revolutionising the management of common cancers, including lung carcinomas^2–4^, head and neck^5^ and skin cancers^6^. The PD-1 molecule, also known as CD279, is a cell surface protein that is expressed by all T cells during activation, and typically serves to down-regulate T cell numbers following virus infections. However, PD-1 can also down-regulate immune responses to cancer, by promoting self-tolerance through suppression of T cell activity. It often shows high and sustained expression levels during persistent antigen encounter in chronic infections and in cancer^7^. PD-L1, also known as CD274, is a ligand for the PD-1 receptor and is commonly expressed on malignant cells. Binding of PD-L1 expressed on tumour cells to its receptor on immune cells can help tumour cells evade antitumour immunity. As circulating anti-tumour cytotoxic T lymphocytes (CTLs) can be detected in a significant proportion of cancer patients, this is considered to be a major mechanism responsible for cancer development and growth. CTLA-4 (cytotoxic T-lymphocyte-associated protein 4), also known as CD152, is another protein receptor that functions as an immune checkpoint. It downregulates immune responses and, like PD-1 and PD-L1, can be targeted by specific ICIs, leading to increased antigen presentation, lymphocyte priming and lymphocyte migration into tumours^8^. Anti-CTLA-4 ICIs are often given in combination with anti-PD-1 or anti-PD-L1 ICIs.

In the earliest clinical trials of ICIs, these agents were employed as last-ditch therapies after the failure of all conventional therapeutic approaches in advanced disease settings. After their efficacy was demonstrated in patients without any effective alternatives, following successful clinical trials, these agents have gradually become accepted as part of first-line therapy of metastatic disease in many settings and their use is currently being explored in the adjuvant setting for high-risk malignancies after definitive local disease control^9^. Despite these successes, many patients do not respond to immunotherapy and a few may even experience acceleration or “hyperprogression” of their disease^10^. Furthermore, a significant proportion of patients will ultimately experience either local or generalised disease progression after promising initial responses.

ICIs and many other immunotherapies are currently extremely expensive and are commonly associated with significant toxicities, especially when given in combination and for a long duration. Therefore, there is an enormous, unmet need to rapidly identify markers of resistance and responsiveness to immunotherapies and define indicators of therapeutic resistance, primary and acquired, both at the level of the whole body and between individual tumours in the same patient. Biopsies currently provide the most useful available biological information when considering ICI for cancer. Analysis of a biopsy can identify and quantify the expression of the immunotherapy target in the tumour and also provide information on the TME. The Immunoscore, which characterizes the complement of immune cell populations, both immunostimulatory and immunosuppressive in biopsy specimens is widely used to obtain information on the TME^11^. However, tumour biopsies provide limited sampling and are impractical to repeat serially. Specifically, they reflect the nature of a tiny part of a single tumour lesion at a single timepoint. Serum biomarkers, including circulating tumour DNA (ctDNA), increase the capacity for defining temporal changes^12,13^, but these generally rely on tractable mutations of biological relevance to the cancer and provide little or no information regarding the spatial distribution of disease and give limited information regarding overall disease burden^14^. They also provide no information on cancer immunity. Conversely, the use of a non-invasive assay of biological targets (be they cancer- or immune-related) using molecular imaging on a whole-body scale with novel positron emission tomography (PET) tracers, has the potential to serially identify heterogeneity of target expression between and within lesions.

Molecular imaging can provide new insights into the spatiotemporal changes that occur in the biological targets of cancer immunotherapies with treatment. Many of these mechanisms remain poorly defined. For example, in vitro studies show that both radiotherapy and chemotherapy can up-regulate PD-L1 expression on tumour cells, potentially converting ICI-unresponsive tumours to being responsive, but this phenomenon is poorly documented clinically. Current clinical practice generally relies on the identification, pre-treatment, of an immunotherapy target on cancer cells. However, in the case of PD-L1 expression, for example, this can often be heterogenous^15^, transient, and powerfully influenced by other cells in the TME^16^. Accordingly, unrepresentative biopsies may lead to the selection of inappropriate therapy. The demonstration of spatial heterogeneity in target expression or variation in that expression over time with molecular imaging provides opportunities to deliver individualised treatment combinations that are based an understanding of the causes of immunotherapy resistance. Indeed, the validity of a radionuclide-based imaging approach to study immunity is already being established in clinical trials^17^, which have confirmed the presence of significant heterogeneity in PD-L1 expression in human tumours. Preliminary results also suggest that appropriate imaging may provide a more robust predictive biomarker than the current biopsy-based assessment of immunohistochemistry or RNA-sequencing^17^. For example, Smit and colleagues found that ^89^Zr-durvalumab (anti PD-L1 antibody) tumour uptake was higher in patients with advanced NSCLC who had a response to durvalumab treatment, but did not correlate with tumour PD-L1 immunohistochemistry^18^. Heterogeneity of target expression between different tumours in the same patient is also of particular interest, given our increasing ability to control lesions that escape from systemic therapy, using targeted local therapies such as stereotactic ablative body radiotherapy (SABR)^19,20^.

Successful immunotherapy depends upon much more than the expression of an appropriate immunotherapy target in a tumour. The cellular componentry, physical architecture and spectrum of bio-active constituents within the TME can all play a vital role in the efficacy of immunotherapy as well as providing additional targets for therapeutic intervention. It is becoming increasingly feasible to image these aspects of the TME and to guide the development of novel therapeutic approaches that will increase the probability of success in the future. Novel PET tracers to image CD8 and other activated T-cells are currently in human clinical trials, and may provide a greater insight into the mechanisms of primary and acquired immunoresistance and hypoprogression^21–23^. A mark of the high level of interest in this area is the rapid development of novel PET tracers to image other components of the immune system^24^ such as CTLA-4^25,26^ and TIGIT^27–29^, where monoclonal antibodies are already in Phase III trials. Other novel immunotherapy targets such as OX40^30^ and B7-H3^31^ are likely to emerge as interesting and useful targets for PET tracer development. At the moment, these remain in the preclinical space, but their translation into human clinical trials will significantly increase our ability to non-invasively characterise the human immunophenotype in vivo.

In this review, we discuss molecular imaging as a promising biomarker to predict therapeutic response to and guide treatment with immunotherapy, with an emphasis on ICI therapies and the TME in which they function. We also outline some of the technical advances that make this approach feasible, but also highlight the challenges facing future clinical trials that would be required to make novel immune imaging technologies accessible in routine clinical practice.

## The patient journey and immunotherapy

### An overview of the current landscape

Lung cancer serves as an illustrative example of the evolving clinical impact of immunotherapy. Non-small cell lung cancer (NSCLC) is the most common cause of cancer-related death world-wide^32^, so initial data showing improvement in survival of patients with advanced disease were greeted enthusiastically and immunotherapies rapidly were incorporated into standards of care^33–35^. Within the last decade, effective therapies for NSCLC expanded to include combined chemoimmunotherapy^36–38^ and combined immunotherapies (i.e. concurrent delivery of more than one immunotherapeutic agent) with combination chemotherapy^39^. ICI was moved earlier in the therapeutic pathway following the landmark PACIFIC randomised trial^3^, which showed that the rate of distant failure in patients treated with potentially-curable unresectable stage III NSCLC treated with definitive chemoradiation was significantly reduced by adjuvant anti-PDL1 antibody therapy given after completion of locoregional therapy, presumably by eradicating micrometastases beyond the radiotherapy treatment volume. In the PACIFIC study, use of ICI was also associated with a very substantial increase in the overall survival rate. Current trials are exploring the role of preoperative ICI in earlier stage, resectable lung cancers^40^. Immunotherapy therefore has the potential to impact the patient journey across the entire NSCLC spectrum, from early stage, through locoregionally-advanced to metastatic disease.

Despite the success of immunotherapy in NSCLC, it is still not clear why some patients respond well and others do not. A wide range of approaches have been explored to develop predictive biomarkers for ICIs, including analyses of PD-L1 or PD-1 expression on tumour cells and infiltrating lymphocytes^41,42^, the pattern of lymphocytic infiltration as reflected in an “immunoscore”^11^, tumour mutational burden^11,43^, microbiome^44^ and consideration of clinical features such as use of steroids and antibiotics. Unfortunately, the mere presence of an ICI target appears insufficient to elicit a successful treatment response. For example, PD-L1 positive tumours that have a “cold” phenotype^45^, do not respond to anti-PD-L1 antibody therapy because blockade of the target, although demonstrably present, does not cause effector immune cells to be recruited to the tumour, or to penetrate its mass to the point that they can engage with individual cancer cells.

Intriguing nuances have emerged in the assessment of lung cancer biomarkers. Although PD-L1 is a useful biomarker in non-oncogene-addicted tumours, it fails to predict benefit in patients harbouring EGFR, ALK or ROS1 genomic alterations, even when PD-L1 is strongly positive^46,47^. Indeed, combinations using tyrosine kinase inhibitors (TKI) with immunotherapy failed to improve on the benefits of TKI alone, despite increased toxicity^48,49^. Accordingly, PD-L1 expression should also be considered in the context both of clinical factors, including tobacco use (associated with higher mutation burden), and molecular status. These correlative findings must have a basis in tumour immunobiology—for example, it may be that oncogene-addicted tumours are not permissive for TIL penetration. Alternatively, the biological effects of PD-L1 expressed on tumour cells or antigen presenting cells (APCs) may be different. Ultimately, imaging approaches that can combine PD-L1, CD8, and an APC marker in a tumour deposit, or, ideally, also in draining lymph nodes, may help to distinguish these possibilities. Even in non-oncogene driven but high PD-L1 expressing tumours, ~70% of patients treated with first line immunotherapy still relapse and die of their disease^50^, underscoring the dynamic role of the tumour microenvironment and the need, not only to better predict the emergence of resistance, but also to develop strategies to abrogate its development. Whilst PD-L1 and CTLA-4 were the first immune checkpoints that were successfully targeted to improve patient outcomes, other molecules are also being explored, either as indicators of resistance such as STK11/TP53 co-mutations^51^ or as targets for immune activation^52^.

### The tumour microenvironment (TME): a largely untapped source of immunotherapy targets

The TME contains many cellular subpopulations that can modulate the response to immune therapy. The most relevant populations include T-cell subtypes, myeloid-derived suppressor cells, macrophages, cancer-associated fibroblasts and cells of the tumour vasculature. Immunosuppressive physiological factors that are frequently present in tumours include hypoxia and acidification of the TME, which have direct effects on lymphocyte function including release of cytotoxic biomolecules such as perforin and granzyme B^53^.

Binding of PD-L1 to PD-1 on the surface of T cells inhibits immunity. PD-L1 exerts its immunosuppressive influence via distinct functions within each immune cell type. In CD8+ cytotoxic T lymphocytes (CTLs) it stimulates apoptosis and exhaustion; conversely, it instigates proliferation in immunosuppressive regulatory T-cells (Tregs)^54^. As monoclonal antibodies targeting PD-L1 and PD-1 and, to a lesser extent, CTLA-4 are the most commonly-used ICIs, it is unsurprising that radiolabelled PET tracers based on these molecules are amongst the first to commence evaluation in clinical trials. Imaging of immune-axis cells, such as CD8 + CTLs and Tregs, could lead to important insights into resistance mechanisms for immune therapy. Identification of an immunosuppressive TME, from which CD8 + CTLs are excluded, or are present but functionally exhausted, could guide the development of future clinical trials, as well as leading to personalised approaches for individual patients. In principle, all of the important cellular and physicochemical properties of the TME can be serially visualized and quantified with PET-imaging^55^.

### Unmet clinical needs

Immune based PET imaging (referred to broadly as Immune-PET in this article rather than immunoPET, which is more widely used in the molecular imaging community to reflect the use of radiolabelled antibodies irrespective of the target) has the potential to fulfil the unmet needs of clinicians for clinically-relevant information to guide decision-making for cancer patients. These questions include; “What is the probability that my patient will respond to a particular immunotherapy?”, “Would monotherapy or combination therapy be most appropriate?”, and “Why has my patient stopped responding to immunotherapy and what can I do about it?”. Timely imaging of specific molecules on the surfaces of neoplastic cells or cells comprising the TME could provide valuable insights into the multiple clinical problems that arise during treatment with immune therapies. The sequential imaging of key immune-related molecules with PET can tell us where these molecules are located, provide semi-quantitative data on their concentration in different tissues and show how they change over time. This global, geographic and evolving perspective is simply unavailable from biopsies or circulating biomarkers.

Whether Immune-PET will be a complementary or competing technology for blood-based methods for monitoring response to immunotherapy is open for debate^56^. Advances in next-generation sequencing (NGS) of circulating tumour DNA (ctDNA) have demonstrated significant capacity to predict the response to immunotherapy in patients with advanced NSCLC^57,58^ and for assessment of minimal residual disease (MRD) burden post-surgery^59^ and immunotherapy^58^. ctDNA is of less utility in early stage NSCLC, with Stage I cancers making up the largest proportion of cancers with undetectable ctDNA^60–62^, either because they do not shed DNA, or do so at levels below the level of detection of the assay. However, the sensitivity of ctDNA can be improved by use of diagnostic tumour biopsy population databases to generate knowledge of tumour-associated mutations, increasing the ability of ctDNA platforms based on panels of commonly involved genes to detect even Stage I disease^59^.

We believe that immunePET has great potential to provide complementary information to liquid biopsy-based approaches, by capturing, lesion by lesion, clonal and spatial heterogeneity. However, this unique integration of the imaging and molecular biomarkers will require the development of radiological standards to capture the complex imaging changes seen during immunotherapy as well as facing a complex validation and regulatory approval pathway.

Beyond the initial selection of therapy, monitoring of response is similarly problematic and has led to modification of the current radiological standard, RECIST^63,64^ to reflect the differing patterns of response now recognized to occur^65^. In addition to the new scheme of iRECIST (RECIST modified for immunotherapy response assessment) proposed for anatomical imaging using CT or MRI^66^, new schemata have also been developed for molecular imaging with ^18^F-fluorodeoxyglucose (FDG) reflecting some of the difficulties posed in monitoring response to ICIs, particularly with respect to early progression and development of new lesions. These include the PET/CT Criteria for Early Prediction of Response to ICI Therapy (PERCRIT)^67^, the Immunotherapy-Modified PERCIST (PERCIMT)^68^ criteria developed in melanoma immunotherapy clinical trials, and the Lymphoma Response to Immunotherapy Criteria (LYRIC) for response assessment in Hodgkin’s lymphoma^69^. Despite these revisions, after treatment, inflammatory cell density and perfusion are often increased, and none of these methods reliably differentiates between “cancer” or “no cancer” regardless of the response scale that is applied to a single FDG-PET scan. Follow-up imaging or biopsy is generally required to differentiate pseudoprogression due to augmented inflammatory cell infiltration of tumour sites from true progression. Following immunotherapy, immunePET scans capable of documenting immune changes could play a clinically relevant role^17^.

**Fig. 1:**
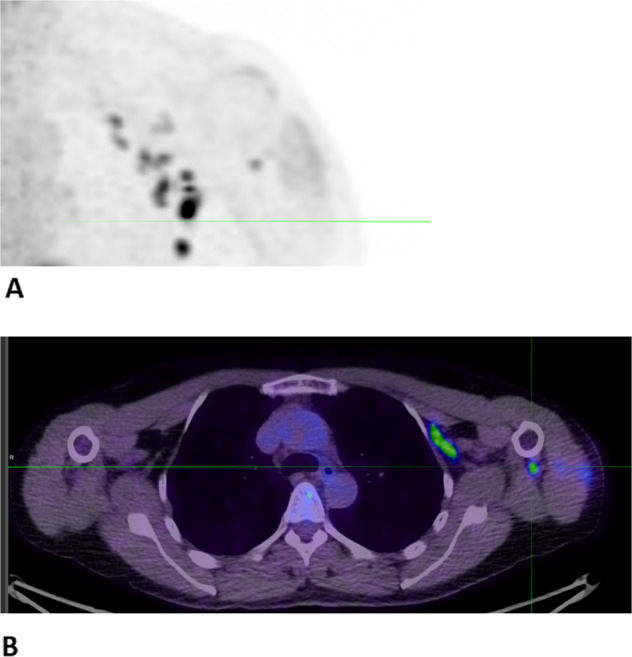
Immune activation imaged by FDG-PET. FDG-PET/CT images of a patient FDG-avid left axillary lymphadenopathy after COVID-19 vaccination, reflecting glucose metabolism in immune cells. In the maximum intensity projection image (panel **A**), lymph nodes appear as black spots and in the transverse PET/CT image (panel **B**) as light green regions.

In addition to suspected early disease progression, another common problem is “stable disease” on immunotherapy, particularly as determined by anatomical imaging. Should therapy be continued or should it be stopped? Anatomically-based imaging cannot inform us whether apparent stability reflects disease control, with replacement of tumour by fibrosis or relatively slow growth of residual tumour. Imaging that reliably distinguishes cancer from non-cancer cells will enable more informed decision-making concerning the timely continuation or cessation of immune therapy, with significant potential benefits in reducing toxicity and cost. There is evidence that a complete metabolic response on FDG-PET/CT may be helpful in this setting^70^. However, when FDG-PET/CT remains positive, differentiating between viable tumour and infiltrating T lymphocytes remains problematic. The distribution of FDG uptake in other tissues, including draining lymph nodes of systemic sites of metastasis or evidence of immune activation in secondary lymphoid tissues such as the spleen^71^ may provide helpful information. Changes in FDG^72^ nodal uptake after anticancer vaccination may be valuable markers of immune response. Such responses are commonly-seen in axillary nodes after COVID-19 vaccination (Fig. 1) and, because they are non-specific, may cause diagnostic uncertainties. In future, immune PET imaging may be used to characterise CD8 T cell/APC migration, for example between tumour and secondary immune organs (typically the site where professional antigen-presenting dendritic cells interact most productively with T cells), and activation status over time, following an immune intervention such as ICI. Persistent localised tumour deposits that have escaped from ICI could be managed with local ablative therapies, while continuing immunotherapy and prolonging the period of benefit.

As indicated above, another challenging problem is the “pseudo-progression”, where lesions increase in size or new lesions appear, consistent with disease progression according to RECIST criteria, but without actual cancer growth^66^. Pseudo-progression is caused by the influx of inflammatory cells and oedema in the tumour. It occurs in up to 20% of patients with metastatic malignant melanoma, but in fewer than 2% of patients with non-small cell lung cancer^73,74^. Metabolic imaging with FDG-PET cannot distinguish true disease progression from pseudo-progression because activated immune cells are FDG-avid^75^. Diffusion-weighted MRI scans also suffer from the same lack of specificity^76^. More sophisticated data-mining approaches using large-scale radiomics of CT and PET scans are unable to accurately differentiate between pseudo progression and real tumour growth^77^. Radiomic approaches have also been unable to usefully predict prognosis^78^ or response to treatment with immunotherapy^79^.

**Fig. 2:**
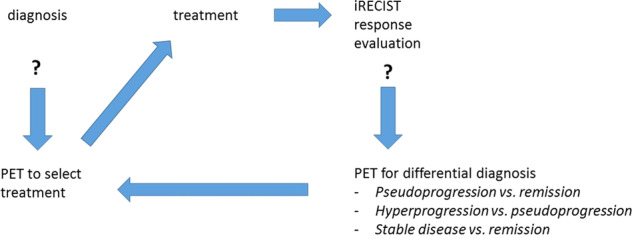
The patient journey: PET in precision medicine. After the diagnosis has been established, PET will help select the optimal treatment to the patient. During the course of the treatment, several questions may arise, which cannot be solved by IRECIST. Here, PET may be indicated to answer specific questions.

The controversial concept of hyper progressive disease has been proposed to describe increased tumour growth and/or spread after the initiation of immune therapy. This is the obverse phenomenon to pseudo progression. Several putative mechanisms have been suggested to explain hyperprogression. In tumours that are abundant in tumour-infiltrating FoxP3^high^, CD45RA^−^, CD4^+^ T cells [effector Treg (eTreg) cells], PD-1 blockade may actually facilitate the proliferation of these highly suppressive PD-1^+^ eTreg cells, resulting in further inhibition of pre-existing antitumor immunity^80^. An imaging method to detect the presence of actively proliferating PD-1^+^ eTreg cells in tumours could therefore be a marker for potentially hyperprogressive cancers. In particular, specific imaging of tumour-infiltrating T-cells by PET scanning may more accurately and quickly resolve these important clinical questions^81^. Hyperprogression may conceivably reflect a state of irreversible CD8 CTL exhaustion. Such T cells typically express high levels of cytotoxins such as granzyme B, but as terminally differentiated cells, typically co-express multiple checkpoint receptors (TIGIT^52^, LAG-3^82^ and TIM-3^83^), have lost their proliferative potential and are close to apoptotic death^10^. By contrast, an effective response to ICI typically involves “epitope spreading”, with recruitment of activated T cells capable of detecting multiple new specificities, as reflected by APC activation in a regional lymph node, and the generation of far greater T cell receptor diversity, both for CD4 and CD8 T cells^84–86^. In Fig. 2, potential roles for Immune-PET at different points of the patient journey are suggested.

## Development of PET tracers to image immunity in cancer

### Designing tracers to image immunotherapy targets

The development of a novel immune-PET tracer presents numerous challenges due to the large number of variables to be considered and optimised. The most widely used ICIs are monoclonal antibodies (mAbs) that bind to CTLA-4, PD-1 or PD-L1. These large molecules are readily-labelled with positron-emitting radioisotopes and PET imaging with the resulting tracers can image in vivo the antigen to which the mAb binds, providing information on both the biodistribution of the antibody and its ability to engage with the target. The high molecular weight of IgG antibodies (~150 kDa) combined with the interactions of their Fc portion with Fcγ receptors mean that antibodies that have a long circulation time, often taking several days to be cleared from the blood and localise in target tissue. One approach to accommodate the long circulation time required to sufficiently distinguish the target from background is to radiolabel the antibody with a positron-emitting radionuclide with radioactive half-life long enough to allow PET imaging several days after injection of the tracer. Clinically useful, long-lived positron emitting radionuclides include ^89^Zr (t_1/2_ = 78.4 h, *E*_*β*+_ 396 keV (23%), *E*_*γ*_ = 909 keV) and ^124^I (t_1/2_ = 4.2 d, *E*_*β*+_ 687, keV (23%). The relatively low positron-emission energies of ^89^Zr results in high resolution images and its 909 KeV γ-rays do not interfere with the detection of 511 keV photons detected in PET imaging, but limit the administered activity that can be used in patients, particularly if serial studies are planned. In contrast, the higher positron-emission energy of iodine-124 can limit the resolution of PET images and the radionuclide also emits a large range of γ-rays with energies sufficiently close to 511 keV that add to background signal on PET images. ^89^Zr can be produced on biomedical cyclotrons by proton irradiation of “natural” ytrrium-89 (^89^Y) targets (of 100% abundance) and this coupled with the lower positron emission energy, and lack of interfering γ-emissions have contributed to it emerging as the radionuclide of choice for radiolabelling full antibodies. However, a potential disadvantage of ^89^Zr is the relatively high-energy γ-emission (909 KeV) which adds to radiation exposure not only to the patient but also to staff members and requires extra shielding for transportation when compared to ^18^F.

Despite the clinical promise of ^89^Zr -labelled full antibodies, the requirement for imaging several days after isotope administration presents some logistical challenges that have the potential to impede their widespread adoption in clinical practice. Nevertheless, initial studies with long-lived tracers, such as ^89^Zr based immune-PET imaging agents, have demonstrated the feasibility and utility of imaging a relevant target and may thereby encourage the subsequent development of alternative tracers based on engineered antibody fragments or receptor avid peptides or small molecules radiolabelled with positron-emitting radionuclides with shorter radioactive half-lives. Within this context, radionuclides of interest include ^68^Ga (t_1/2_ = 68 min, *E*_*β+*_ = 830 keV 89%)^87^, ^64^Cu (t_1/2_ = 12.7 h, *E*_*β+*_ = 278 keV 19%, *E*_*β−*_ = 190 keV 39%, EC 61%)^88,89^ and ^18^F (t_1/2_ = 110 min, *E*_*β+*_ = 250 keV)^90^. Such smaller molecule tracers have the potential to rapidly characterise dynamic changes in immunotherapy targets^87,91^. For example, we know that CTLA-4 expression rises rapidly and transiently after radiotherapy. This expression lasts only a few hours, and is most strongly induced by hypofractionated radiotherapy^92,93^. Imaging based on a radioisotope with a shorter half-life could visualise short-lived changes in patients resulting from treatment. Shorter half-life isotopes may also be useful for screening patients for mAb-based theranostic treatments with immunotherapy-linked, DNA-damaging radioisotopes^94^. A further advantage of short-lived isotopes is that patients may potentially be screened for the same immune and metabolic parameters on multiple successive occasions, to track the quality and magnitude of the immune response “in real time”.

For each combination of radioisotope and targeting molecule selected for development, various chemical methods of bioconjugation are available that can affect performance of the resulting imaging agent. Even small modifications to large proteins can drastically affect their in vivo properties. When optimising the administration of antibody-based tracers, the specific activity and amount of tracer injected are important considerations to reduce off-target uptake by saturating both off-target antigen (e.g., PD-L1) and FcRy receptors^95^. A detailed review of these important considerations when radiolabelling targeting agents such as antibodies is available^96^.

For clinical use, longer-lived radioisotopes can be manufactured off-site, under conditions of good-manufacturing practice (GMP), and transported to the patient, without undergoing significant decay. They can facilitate multiple test-retest imaging studies per injection. In contrast, short-lived tracers generally require synthesis on-site by an experienced radiochemist and may require an in-house cyclotron and are more challenging to comply with any GMP requirements that might apply. As suggested above, successful initial studies with long-lived tracers, such as ^89^Zr based immune-PET imaging agents, may encourage the subsequent development of shorter-lived radiotracers, based on radioisotopes such as ^18^F that provide more timely images and avoid the radiation protection issues associated with the longer half-life isotopes. With any new tracer, standardisation of image acquisition and assessment parameters is vital to enable accurate inter- and intra-patient comparisons. Table 1 provides a broad overview of selected immunotherapy targets and relevant methods of radioisotope imaging that have been investigated to date and Table 2 lists selected studies of PET Tracers of CD8, PD-1 and PD-L1 that have entered human clinical trials.

**Table 1:** Methodologies and radioisotopes used to image immunotherapy targets.

Immunotherapy Type	Molecular Imaging Target	Tracer Type	Applicable Radionuclide Used (reference in brackets)
Anti-PD-1 Antibody	PD-1 Receptor	mAB	^89^Zr^64,114,115,130^ Cu^88,113,131–133^
Anti PD-L1 Antibody	PD-1 Ligand	mAb	^89^Zr^17,134–142^ ^64^Cu^88,89,132,111,143^ In^144^
		Probody	^89^Zr^142^
		Peptide	^18^F^145^ ^68^Ga^146^ ^64^Cu^144,145,147^
		Affibody	^18^F^127^
		Adnectin	^18^F^116,128,148^
		HAC-PD1	^64^Cu^87,131^
		Nanobody	^99m^Tc^68,97^ Ga^149^, ^18^F^150^ ^64^Cu^150^
		(Camelid VHH)	^68^Ga^151^
		Single Domain antibody	^89^Zr^152,153^
Anti CTLA-4 Antibody	CTLA-4	mAb	^64^Cu^25,26,154^
	CD28, CD80, CD86	F(ab’)2 fragment	^64^Cu^26^
TIGIT Antibody	TIGIT protein extracellular domain	mAb	^64^Cu^29^ ^89^Zr^29^
LAG-3 Antibody^155,156^	LAG-3 protein extracellular domain	Nanobodies	^99m^Tc^157^
Cellular Therapies			
CAR T-Cell Therapy	Direct labelling CAR T-cell	^18^F-FDG	^18^F^158,159^
		^64^Cu-PTSM	^64^Cu^160^
		SPION-^64^Cu	^64^Cu^161^^*^
		^89^Zr-Oxine	^89^Zr^126,162–165^
		^89^Zr-DBN	^89^Zr^166^
		^89^Zr Df-Bz-NCS	^89^Zr^167^
		Minibody	^89^Zr^22^
		Bispecific antibody	^89^Zr^168^
		Antibody Fragments	^89^Zr^169^
		Sodium Iodide Symporter	^124^I^169^
	Reporter Gene Labelling Transporter	Norepinephrine transporter	^18^F^170,171^
	Enzyme	Pyruvate Kinase	^124^I^172^
	Cell Surface Receptor	Thymidine Kinase	^18^F^173^
		Somatostatin receptor	^18^F^174^ ^68^Ga^175^
	Cell Surface Protein	PSMA	^18^F^176^
Immune Cell Subtype(s)			
Activated CD8+T lymphocytes	dCK, dGK	F-AraG	^18^F^177^
CD3+T Lymphocytes	CD3	mAb	^89^Zr^178,179^
		Bispecific antibodies	^89^Zr^180,181^
Cytotoxic T Lymphocytes & NK Cells	Granzyme B	NOTA-GZP	^68^Ga^168,178,182^
Tumour Associated Macrophages (TAMS)	High density lipoprotein	Phospholipid or ApoA1 HDL	^89^Zr^183^
	Macrophage mannose receptor (MMR) or CD206	Anti-MMR single domain Ab fragment	^68^Ga^184^
	TSPO	18 kDa (TSPO) transmembrane domain protein on the outer membrane of mitochondria	^18^F^185,186^
	Macrin	Polyglucose nanoparticle	^64^Cu^187^
	Liposomes	Mannose coated liposomes	^64^Cu^188^
	CD11	Monoclonal Antibody	^89^Zr^189^
Myeloid Derived Suppressor Cells	CD11	Monoclonal Antibody	^89^Zr^189^
Cancer Associated Fibroblasts	Fibroblast activation protein		^68^Ga^190^
	Fibroblast activation protein inhibitor		^18^F^191^ ^68^Ga^192^

*mAB* monoclonal antibodies, *HAC* high-affinity consensus, *bsAb* bispecific antibodies, *CAR* Chimeric antigen receptor T-cell, *dGk* Deoxyguanosine kinase, ^*64*^*Cu-PTSM* ^64^Cu-pyruvaldehyde-bis(N4-methylthiosemi-carbazone), *SPION* super paramagnetic Iron-Oxide nanoparticles, ^*89*^*Zr r-DBN* ^89^Zr -desferrioxamine-NCS, ^*89*^*Zr Df-Bz-NCS* ^89^Zr-p-Isothiocyanatobenzyl-desferrioxamine.

*Denotes MRI imaging.

**Table 2:** Selected Studies of PET Tracers of CD8, PD-1 and PD-L1 in Human Clinical Trials.

	Molecule	Trial Details
PD-1		
	^89^Zr-DFO-Nivolumab	13 patient study in advanced NSCLC. Dual tracer study with ^18^F-BMS-986192. IHC PD-1 and PD-L1 staining and SUVpeak for ^89^Zr-nivolumab and ^18^F-BMS- 986192 is strongly correlated (*Rs* = 0.68, *p* < 0.0001 Spearman rank correlation). Lesions with no PD-1 expression in aggregates have a lower ^89^Zr-nivolumab SUVpeak; *p* = 0.03^116^.
	^89^Zr-DFO-Pembrolizumab	12 patients in advanced NSCLC. Tracer seen in 47% of tumour lesions correlated with pembrolizumab response, but did not correlate with PD-L1 or PD1 IHC^193^.
PD-L1		
	^89^Zr-DFO-Atezolizumab	FTIH 25 patient study. Pretreatment atezolizumab-PET better correlated with Immunotherapy response than IHC or RNA-seq markers^17^.
	^18^F-BMS-986192	^18^F-BMS-986192 SUVpeak is higher in patients with ≥50% tumor PD-L1 expression. (*p* = 0.018), SUVpeak of the ^18^F-BMS-986192 tracer is higher in responding lesions as compared to non-responding lesions (*p* = 0.02 Mann–Whitney U-test).
	^89^Zr-DFO-Durvalumab	RaDD Study - currently recruiting^21^. ^89^Zr-durva PET during chemo/RT for DLBCL. Trial Registration ClinicalTrials. gov Identifier: NCT03107663^194^. No significant toxicity reported in >18 patients. NKI 13 patient study in patients eligible for 2nd line ICI. ^89^Zr-durva correlated with disease response but not PD-L1 IHC^18^.
	^89^Zr-DFO-Sq-Durvalumab	ImmunoPET study – currently recruiting. ^89^Zr-durva during concurrent chemoRT in Stage III NSCLC. Australian Clinical Trial Registry: ACTRN12621000171819^195^.
CD8 T-cells		
	^89^Zr-DFO-IAB22M2C minibody	FTIH 6 patient study: uptake in tumour lesions peaked at 24 h^21^. Ph II (mixed histology) 15 patient study – increased tracer uptake noted in patients 28 days post-immunotherapy^22^. RaDD study in B Cell lymphomas now recruiting^194^. No significant toxicity yet documented.
Activated T-cells		
	^18^F-AraG (Arabinofuranosylguanine)	FTIH 6 patient study with no significant adverse events seen^21,23^. Ongoing studies in 18 patients with no drug related adverse events. (Unpublished data supplied by Cellsight Technologies).

### Practical issues in developing tracers to image immunotherapy targets

An ideal imaging tracer for an immunotherapy target would have high specific uptake and retention on, or in the cells that express the target and exhibit rapid blood clearance, providing high-resolution images with excellent target to background ratios at convenient time-points following administration. Image resolution is modality dependent (e.g., higher for PET vs SPECT), instrument capabilities and the physical half-life of the isotope used. Many promising positron-emitting radiotracers have been evaluated. Representative examples of some of these radioisotopes are given in Table 3. Whilst the long-lived isotopes (^89^Zr, ^124^I, ^64^Cu) are suitable for the antibody-based imaging agents discussed above, shorter half-life isotopes (^68^Ga, ^18^F) are better suited to peptide and other small molecule-based imaging that clear more rapidly from the blood. Binding of metal-based radionuclides to targeting agents is achieved attaching a bifunctional chelator to the antibody, which can bind the metal ion. The selection of chelator is dependent on the radionuclide used and it is essential that this radioactive metal chelate complex is stable in vivo, as leakage of the metal from the antibody-chelate complex leads to increased background signal and dramatically reduces the quality of the PET image. The method of chelator attachment, along with the number of chelators attached to the targeting agent, can greatly influence the stability and in vivo biodistribution of the tracer. This is due to changes in ionic charge and isoelectric point of the tracer, blockage of the targeting agent at the site of antigen binding, and aggregation (the formation of dimers, trimers and oligomers, which can reduce target affinity and cause precipitation). These properties can be challenging to predict and must be optimised during preclinical development. Degradation of peptides in vivo by serum peptidases is also an issue for many peptide-based targeting agents even when high binding ability is demonstrated in cell culture and this can limit clinical translation.

**Table 3:** A selection of available positron emitting radioisotopes with potential for Immune-PET imaging.

Isotope	Half-life	Production	Advantages	Disadvantages
^18^F	108 min	Cyclotron	Best for small molecule targeting agents.	Half-life not long enough for most Abs, challenging chemistry for attachment to Abs.
			Widely available for clinical use.	Half-life too short for production off-site and transport
^68^Ga	68 min	Generator	Isotope production is available from a portable generator available at most large imaging centres.	Half-life too short for production off-site and transport.
				The ^68^Ge source for the generators can be scarce.
				Local generator shortages have occurred.
^64^Cu	12 h	Cyclotron	^67^Cu theranostic pair. Mid-range half-life makes it suitable for a wide variety of targeting agents.	Not widely available for clinical use.
^89^Zr	78 h	Cyclotron	Long half-life best for antibody-based imaging agents.	High-energy gamma emissions; radioprotection must be considered. Suitable for centralized production and distribution.
			Tracers can be produced off-site and transported.	Radiolysis often a problem.
^124^I	4.2 days	Cyclotron	Long half-life best for antibody-based imaging agents.	High-energy emissions leading to low resolution.
			Tracers can be produced off-site and transported.	Undergoes dehalogenation in vivo.
				Not widely available for clinical use.

By far the most commonly used PET tracer in oncology is ^18^F, (used in ^18^F-FDG for metabolic imaging), a cyclotron-generated isotope with a short half-life of 108 min. To enable the use of ^18^F for imaging immunotherapy targets, smaller molecules than mAbs have been investigated, including peptides, proteins or antibody alternatives such as nanobodies and affibodies.

“Small-format” antigen ligands have the advantage of rapid clearance, and can potentially be engineered as bispecific agents, or with epitope tags that may serve as signal amplifiers. An illustrative example of a peptide tracer is the cyclic 15-amino acid WL12, which was selected from a library of peptides shown to specifically bind to PD-L1. Molecular docking experiments suggest that WL12 binds to a region of PD-L1 that overlaps with the binding sites for the PDL1 antibodies atezolizumab, avelumab and durvalumab. The peptide was conjugated to a macrocyclic chelator, NOTAGA, and radiolabelled with ^64^Cu. The resulting tracer was evaluated in multiple mouse xenograft models using PET imaging. The [^64^Cu]CuWL12 tracer was able to image variable PD-L1 expression in xenografted tumours. Compared to radiolabelled IgG, the fast clearance of peptide enabled images with high uptake and low background to be obtained only 120 min post injection. Nanobodies (small antigen binding fragments of camelid antibodies, 15 kDa) have been assessed in SPECT imaging after labelling with ^99m^Tc^97^.

An alternative experimental approach, that enables antibodies to be labelled with short half-life positron emitting isotopes involves “pretargeting”, in which the mAbs are chemically modified to contain a functional group that selectively reacts with a specifically-designed radiolabelled reaction partner that is administered after the mAb has had time to become bound to the target^98–100^. Pretargeting approaches have been used for PET imaging in relapsed medullary thyroid cancer, where high tumour uptake and contrast have been observed using pretargeted anti-CEA immuno-PET^101^. The short-lived positron emitter, ^68^Ga^102–104^, is available from a generator rather than a cyclotron, thereby increasing its availability. Like ^18^F, its short half-life of only 68 minutes limits it to non mAb-based tracers, unless pretargeting is used. Although not specifically applied to imaging immune targets, an approach using non-radioactive bispecific antibodies followed by clearing of the blood using a hapten approach and then a second radiolabelled agent that binds to the pretargeted antibody has been described^105^, aiming to increase target to background contrast and enhance diagnostic sensitivity.

For mAb-based PET imaging, the long-lived positron emitter ^124^I is rarely used due to dehalogenation of mAbs in vivo and therefore, ^64^Cu and ^89^Zr are currently considered the most suitable available choices. As well as facilitating imaging, pre-targeting approaches can also facilitate the identification of theranostic radiotherapy targets, thus providing prospective dosimetry from the imaging tracer for the therapeutic radioimmunoconjugate^98–100^. Several imaging isotopes have suitable theranostic “pairs”. For example, ^89^Zr can be paired with the therapeutic radioisotope, ^177^Lu, to both image and therapeutically irradiate targets. ^64^Cu has a shorter half-life than ^89^Zr and the imaging and therapeutic radionuclide pair ^64^Cu/^67^Cu can be used in a true “theranostic” approach^106^.

## Translation into clinical practice

### Imaging the cellular tumour microenvironment

Tumours that respond to immune checkpoint inhibition typically have a distinctive TME, with a rich infiltrate of T-cells penetrating deep within the tumour (not excluded entirely, or restricted to its periphery) and in the surrounding stroma^107,108^. These suppressed T-cells are “exhausted” by chronic exposure to cancer-induced immune checkpoints^109,110^, and are therefore likely to respond well to ICI. By reversing the suppression within this state of adaptive immune resistance, these immunotherapies permit activation of immune cells, including CD8 + CTL’s, which play a key role in cancer cytotoxicity. Conversely, immunotherapy resistance can develop if any of these factors are missing^111^.

As we have seen, the mere presence of an immunotherapy target in a tumour does not guarantee a therapeutic response as multiple other factors can influence clinical outcome. For example, radiation-induced pro-immunogenic responses are likely to be dictated by tumour type, tissue niche and TME, and are also influenced by dose and fractionation. In AT-3-OVA mammary tumours, mildly hypo-fractionated radiotherapy could evoke the anti-tumour activity of CD8 + T-cells. However, such responses were not supported by high dose radiotherapy, largely due to the induction of immunosuppressive Treg responses^93,112^. This complexity makes it prohibitively expensive to define the optimal treatment approach for each tumour type through clinical trials. However, the establishment of a process to test potential imaging biomarkers using rapid preclinical validation in animal models may enable us to streamline the clinical implementation of novel immunotherapy tracers.

### Animal studies: opportunities and limitations

**Fig. 3:**
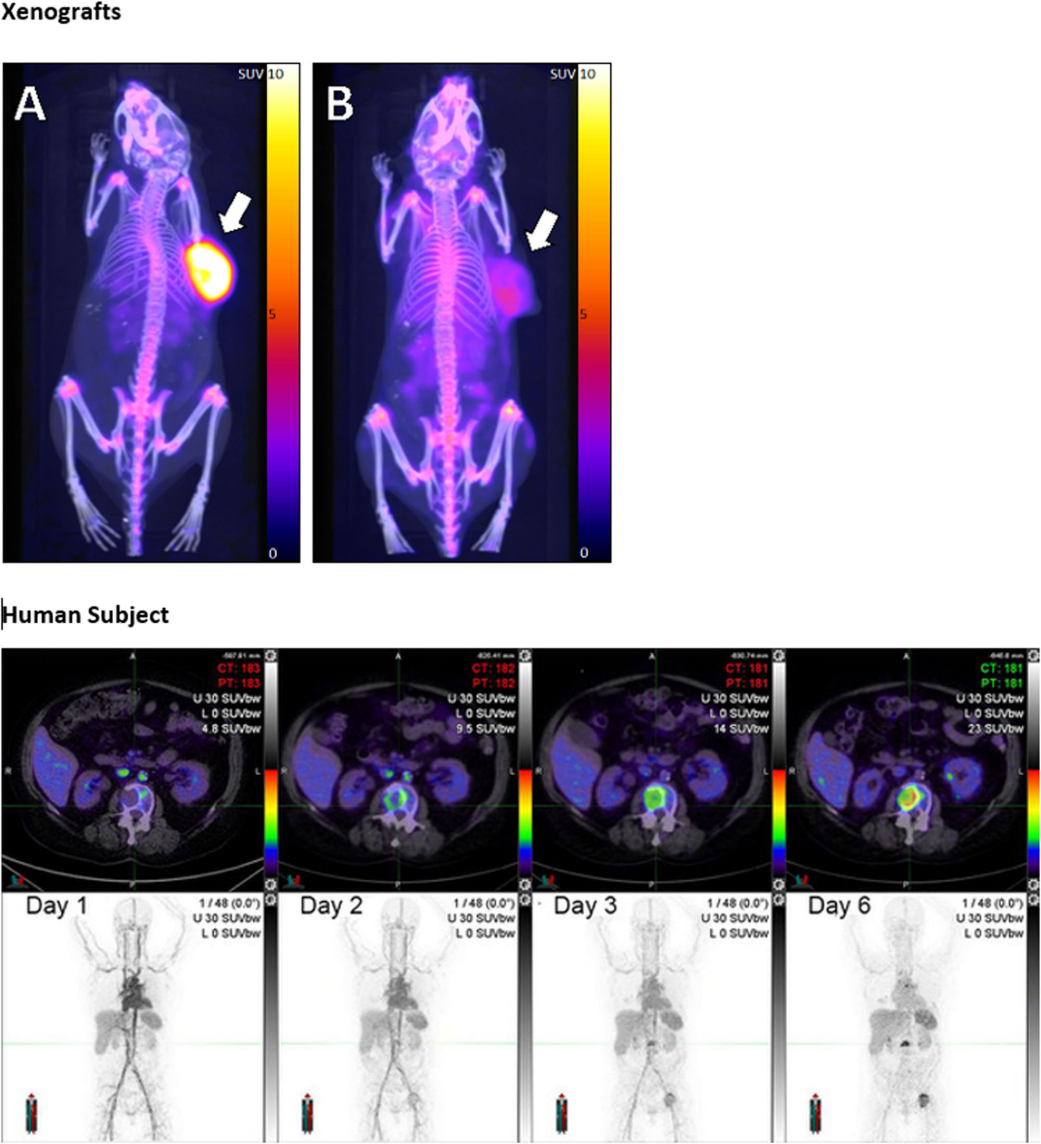
Imaging PD-L1 in xenograft models and in a human subject using ^89^Zr-DFO-Sq-Durvalumab PET/CT. The upper panels show ^89^Zr-DFO-Sq -Durvalumab PET/CT imaging of NSG mouse bearing either HCC-827 PD-L1 high tumour (Panel **A**) or a low-to-non PD-L1 expressing A549 tumour (Panel **B**) at 144 h after PET tracer injection. Pseudo-coloured SUV overlays on CT scan maximum projection images are shown. Subcutaneous human tumour xenografts on right flanks are indicated by arrows. The lower panels show the same ^89^Zr-durvalumab, as validated in the xenograft model, used for PET/CT imaging in a human subject with PD-L1 positive (90%) stage IV NSCLC. Over 6 days, sequential images show gradual accumulation of tracer in distant metastases. Axial PET/CT images (upper panels) show increasingly intensification of uptake in a vertebral body metastasis. Maximum intensity projection (MIP) images (lower panels) show blood pool imaging (day 1) and then gradual accumulation in metastatic disease, including lumbar spine and left hip. (Images courtesy of Dr Tim Akhurst, Peter MacCallum Cancer Centre).

Before being tested in humans, candidate Immune-PET tracers are typically evaluated in mice. For example, human xenografts in immunodeficient mice can be used to investigate the ability of new tracers to survive in the circulation and achieve binding to targets on tumour cells in vivo, in preparation for human use (Fig. 3). Limitations of these animal models are encountered both in the use of human xenografts in immunocompromised mice and murine tumours in immunocompetent mice. If the immune response to human cancer is compromised by suppressive mechanisms raised only in an immunocompetent host, the use of immune-compromised mice may be uninformative. Despite the drawbacks of these models, some common trends have been observed that have informed the development of human tracers. For example, hepatic clearance of mAbs results in high liver uptake and retention for all mAb-based tracers, and high expression of PD-1 and PD-L1 in healthy spleen and lymph nodes results in specific binding and high uptake in these regions. Target-specific uptake can be effectively blocked by the administration of a low dose of cold (non-radiolabelled) mAb, a commonly used strategy in when imaging mAbs. Off-target uptake due to expression of the target antigen in other organs or due to interactions between the Fc portion of the mAb with endogenous FCyR may need to be saturated prior to administration of the tracer.

Preclinical work with a ^64^Cu-DOTA labelled *anti* –PD-1 mAb (RMP1-14) and *anti* –PD-L1 mAb ^64^Cu-DOTA-10F.9G2 demonstrated that changes in PD-1/PD-L1 expression could be detected following treatment with combined immunoradiotherapy, and that lung tissue exhibited IFN-γ-inducible PD-L1 expression^88^. The RMP1-14 tracer (a hamster mAb raised against mouse PD1 and labelled with ^64^Cu-DOTA)^113^, ^89^Zr-Df labelled pembrolizumab^114^, and ^89^Zr-Df labelled nivolumab^115^, have all demonstrated similar specific and non-specific biodistributions in the liver, spleen and lymph nodes in preclinical models.

^89^Zr-nivolumab uptake in lymphoid organs was blocked by the administration of (≥1 mg/kg) of cold mAb in cynomolgus monkeys. When translated into human studies, this tracer gave promising results, with tumour SUVpeak values correlating with PD1 tumour expression assessed by IHC of tumour. Tumour uptake also correlated with therapeutic response to nivolumab on post treatment imaging. Optimum imaging was at 160 h post administration, and consistent with preclinical studies spleen and liver uptake were observed. Some lesions had high tracer uptake, despite apparently low expression by IHC, probably a result of tumour heterogeneity. As in the previously noted lung cancer study^18^, PET imaging had the potential to predict patient response better than IHC, although this needs further investigation in larger studies^116^.

In animals, tracers using *anti* –PD-L1 mAbs have been investigated more comprehensively than those for PD-1 and have shown great promise, with generally consistent biodistribution in preclinical models. Uptake in the liver, spleen, lymph nodes, and brown adipose tissue (due to PDL1 expression on CD45 + leukocytes) are commonly observed, and in some case in lung and thymus^88,89,91,117–125^. Tumour uptake between each study varies greatly, likely due to the differences in the murine models, tumour types, and mouse/human cross reactivity differences in the antibodies tested. Some notable findings include the detection of significantly higher PDL1 expression in tumour-derived 4T1 cells than in cultured cells^89^. ^64^CuDOTAGA-atezolizumab was able to delineate PDL1-positive induced lung metastases of a PDL1 expressing MDAMB231 tumour^126^, and ^89^Zr-DF-atezolizumab visualised increased PDL1 expression following external beam radiation, along with increased bone uptake particularly within the radiotherapy field^122,125^.

In recent years, alternatives to full IgG mAbs have been investigated in animal systems as PD-L1 targeting agents, often in an attempt to reduce the time required between administration and optimal imaging. These include heavy chain camelid antibodies such as ^89^ZrDf-KN035. A high-affinity consensus protein (HAC) was labelled with ^64^CuNOTA, ^64^CuDOTA, ^68^GaNOTA & ^68^GaDOTA, with each demonstrating different distribution profiles and highlighting the importance of radioisotope and chelator selection. This protein allowed imaging at 1 h in pilot mice studies^87^. Another short timepoint protein-based tracer has also reported, a anti -PDL1 affibody (58-amino-acid scaffold protein with PDL1 binder labelled with NOTA-Al^18^F which underwent renal clearance and allowed imaging 30–90 min after administration. However, these small antibody-alternatives tend to have hepatic or renal clearance^45,127^, as well as some uptake in non-target normal tissues such as salivary glands^45^. They are, despite these drawbacks, the subject of intense development to optimise their pharmacokinetics.

Perhaps the most promising of the alternative antibody-based tracers, with early human data, is the adnectin ^18^F-18F-BMS-986192 (an engineered, target-binding protein with low amino acid similarity, but which folds similarly to an antibody-variable domain, approx. 10 kDa). Tumour uptake is achieved 90 min post administration in mice, and cynomolgus imaging revealed renal clearance made the kidney the dose-limiting organ. In NSCLC patients, imaging at 1 h post administration visualised PDL1-positive tumours and SUVpeak uptake correlated with expression later confirmed by IHC. The tracer uptake was also correlated with response to nivolumab treatment following the imaging study^116,128^.

### Future clinical trials and therapeutic approaches

Murine studies provide a treasure trove of biological data to help inform the future clinical trials needed to improve prediction or assessment of immunotherapy response. For example, sequential combinations of PET tracers, in suitable animal models, could feasibly image in vivo CD8+ T-cell infiltration, CD8+ activation and ICI binding. This could determine whether immunotherapy resistance is due to a hostile immune phenotype (immune-desert where there is little infiltration of CD8+ cells within the tumour; or immune-excluded, where the T cells are confined to the extreme periphery of a tumour mass) or T-cell exhaustion. Such studies would help select promising tracers or combinations of tracers for human trials and could suggest potentially cost-effective imaging schedules.

**Fig. 4:**
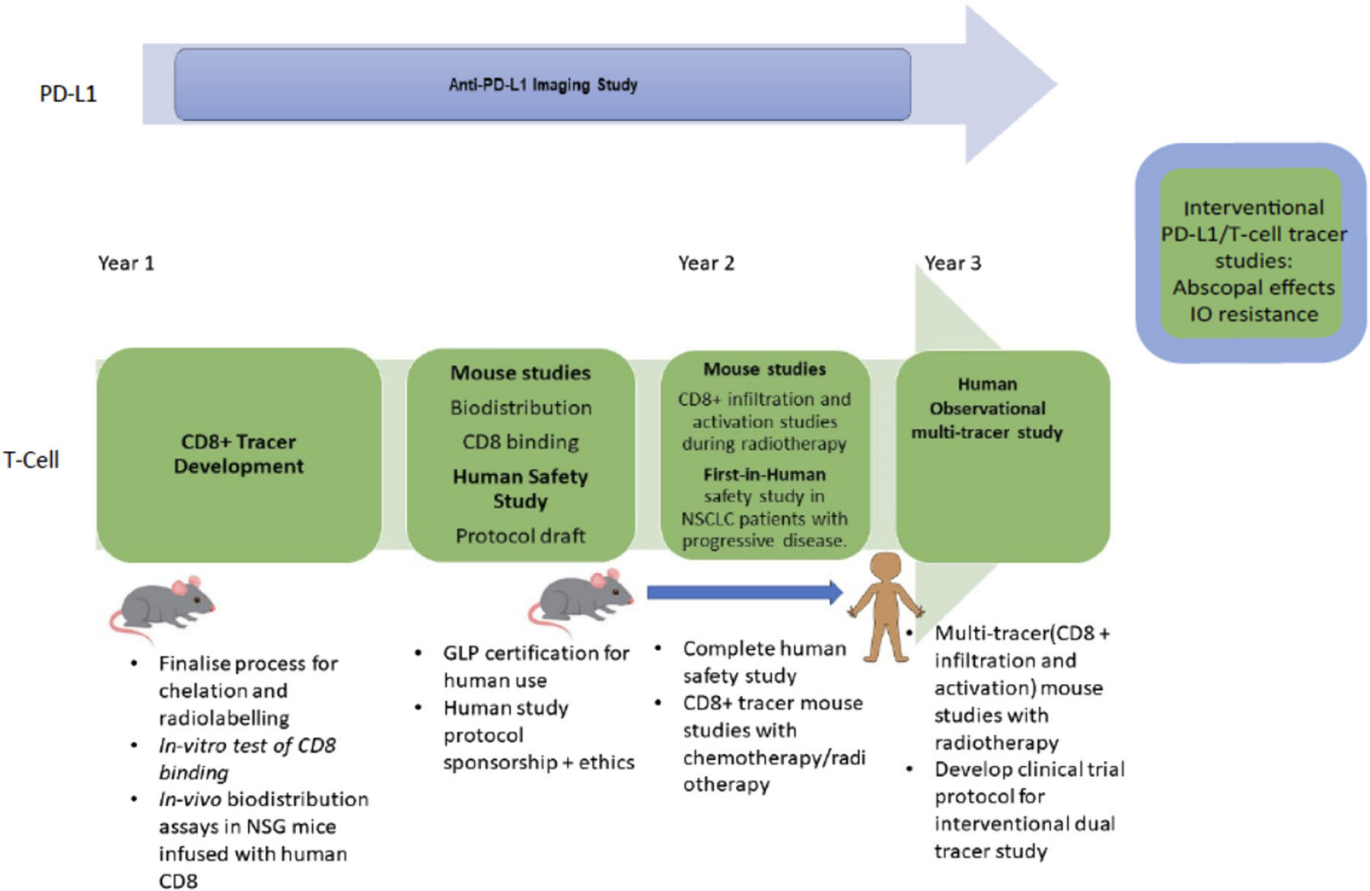
Development of a putative novel tracer. Novel tracer development of a putative CD8 tracer, based on small-antibody alternatives, which could then be integrated with existing tracers to image activated CD-8 cells, such as ^18^F- arabino-furanosylguanine (ARA-G). Combination studies with T-cell tracers and tracers for PD-L1/PD-1 such as adnectin ^18^F-18F-BMS-986192 could help determine the causes of immunotherapy resistance in patients on ICI, as well as being used to investigate the mechanisms of therapeutic strategies such as induction of abscopal effects after radiotherapy.

**Fig. 5:**
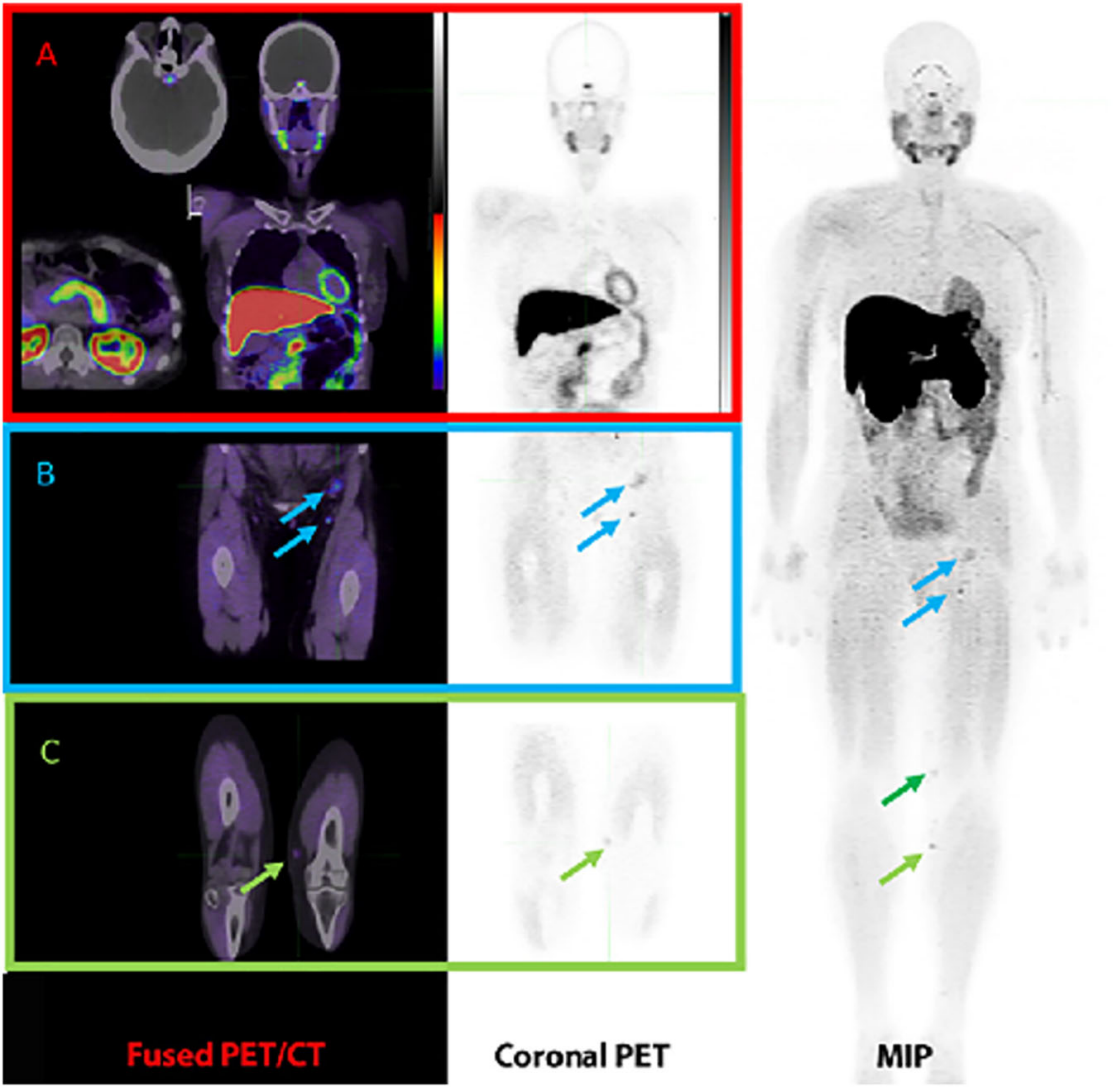
Imaging activated CD8 T cells with ^18^F-AraG. Baseline imaging a patient with recurrent malignant melanoma, scanned at 60 min after IV injection of approximately 185 MBq of ^18^F-AraG prior to immune check-point therapy. A Maximum intensity projection (MIP) image demonstrates uptake in inguinal nodal disease (Blue arrows) and in transit nodules (Green arrows). Panel (**A**): Fused PET/CT and coronal PET images demonstrate moderately intense uptake in the pituitary, salivary glands, myocardium, liver and pancreas. All these organs are subject to immune-related adverse events and how this biodistribution will impact diagnostic performance is unclear. Panel (**B**): Increased uptake is observed in two inguinal lymph nodes, with associated nodal enlargement of the upper node. Panel (**C**): The small lower in transit nodule is clearly identified on both PET and correlative CT (Image provided by Dr Diwakar Davar and Dr Shyam Srinivas from University of Pennsylvania Medical Center and Dr Jelena Levi of CellSight).

In Fig. 4, we outline a possible clinical trial where the short-lived radioisotopes ^68^Ga and ^18^F are used to image CD8+ T-cells and to demonstrate CD8+ activation. Similar tracers are already available for human research (Fig. 5), and increasingly will be clinically accessible (Table 2). Initial studies in mouse models would demonstrate their ability to bind to the relevant targets in vivo. Subsequent immunotherapy imaging clinical trials in humans, informed by data from murine models, would be designed, with full awareness of the limitations of the pre-clinical animal systems, both in terms of biology and of scale. In routine clinical practice, it will be impractical to use multiple tracers because of factors such as cost, radiation dose and regulatory approval. Concentrating research on areas of currently unmet clinical need at critical treatment-decision nexus points would be the most promising approach to developing cost effective and useful immune PET imaging. For example, differentiating pseudoprogression from hyperprogression, or deciding whether ICI could be withdrawn in cases of immune-related adverse events after an abbreviated course of ICI therapy where this apparent residual disease on anatomical imaging. Head-to-head comparison of competing tracers in such research settings can be ethically and logistically challenging in unwell cancer patients imaged in busy molecular imaging facilities. Sophisticated radiochemistry production capabilities are often needed for novel tracers. Accordingly, parallel studies using single agents paired with more widely available FDG PET/CT may represent the most practical development pathway in humans. An important component of any such trials should be evaluation of the impact and appropriateness of information on decision-making and of the cost implications on “whole-of-care”, which includes not only the cost of the imaging paradigm but also the ability to avoid the costs of continuing ineffective therapy or prematurely ceasing treatment in patients with an incomplete response. Should a novel immune imaging agent be found to be indispensable in a clinical setting, upon entering routine clinical practice, costs would rapidly decline due to economies of scale and efficient production and distribution.

As a general template, in clinical trials where it is important to image biomarkers, the “imaging biomarker roadmap” should be followed^129^. A clear strategy will be needed to implement the use of PET imaging of the immune system in clinical practice, in order to bridge the translational gap between pre-clinical and clinical experiments. When a promising imaging tracer has passed pre-clinical and toxicity evaluations and enters initial clinical trials, it is important to be clear on the purpose of the study. For example, is the tracer likely to deliver greater biological insight or better prognostication for the individual patients? Alternatively, is the aim to select the best treatment or predict of response to a particular agent?

Significant challenges must be overcome before full clinical implementation of immune-PET can be achieved. The therapeutic landscape will continue to evolve rapidly, adding an increasing array of novel individual and combination therapies, all requiring evaluation in multiple clinical trials. To keep pace with this therapeutic pipeline, an equally efficient pipeline for companion diagnostic development is required. Combinations of immunotherapeutic agents with chemotherapy, targeted agents and radiotherapy as well as multiple variations in immunotherapy agents themselves may also impact the predictive value of both generic and specific companion diagnostic agents. In some combinations, the response may primarily be due to non-immunological mechanisms. The current standard of care for therapeutic response assessment, of anatomical imaging with CT or MRI, increasingly enhanced or supplanted by use of FDG PET/CT, is well established. Significant incremental benefit must be demonstrated before immune-PET agents could supplant or even replace existing imaging modalities. Therefore, in the first phase of development, demonstration of efficacy of the novel imaging approach as a predictive biomarker of response, particularly in monotherapy or combination ICI without addition of conventional therapies, would be most useful. Concurrent translational biomarker correlations, including lesion and liquid biopsies where available, could both help validate the scan findings and demonstrate any incremental diagnostic utility. Further studies could focus on situations where there is uncertainty with respect to continuing or ceasing ICI therapy based on conventional imaging response assessment.

## Conclusion: new treatment approaches based on immune-PET

Fully developed immune-PET, able to non-invasively characterise immune responses to cancer and anti-cancer therapies, would provide a powerful tool to identify specific in vivo diagnostic features specifying which mechanisms of immune-evasion are operative in that particular patient. This critical information would then inform the selection and tailoring of single or combination therapies most likely to abrogate these mechanisms and provide the best chance of a therapeutic response.

We are in desperate need of biomarkers that can predict which patients are most likely (or least likely) to respond to immunotherapy. In this article we have focused on established approaches to solid tumour immunotherapy with immune checkpoint blockade, but the need for predictive immune imaging is even more pressing for some of the more novel therapeutic approaches. For example, when chimeric antigen receptor (CAR) T cell therapy is used against a validated target such as CD19, responsiveness in a disease such as diffuse large B cell lymphoma is unpredictable, and failure becomes more likely as the patient’s age increases. Thoughtfully designed clinical trials that integrate Immune-PET as an in vivo, real-time biomarker may help fill this critical gap and identify patients most suitable for this revolutionary but highly expensive therapy.

Optimal utilisation of immunotherapies in patients with stable disease is also a key clinical area where the use of Immune-PET could be enormously helpful. The implementation of therapeutic monitoring with Immune-PET could identify patients with no evidence of active disease or with only minimal residual disease, and could allow them to cease treatment, thus avoiding the clinical toxicity of unnecessary, ongoing immunotherapy. The potential for so-called “financial toxicity” to patients and health care systems is a significant drawback of most currently available ICI agents. In such settings, a single immune PET scan could be highly cost-effective.

As we have shown, immune-PET tracers are clinical tools that may, in the near future, be used to provide valid surrogates of response. For example, Immune PET may demonstrate recruitment of the TILs that will induce a therapeutic response. Conversely the imaging might demonstrate a suppressive cellular response, or show that the recruited T-cells are activated or exhausted, and indeed indicate if any potential targets for immune therapies are present.

Immune-PET has the potential to transport us into an era where we can make assessments about the likely efficacy of ICI soon after treatment commences, enabling early cessation of therapy if required. The capacity of Immune PET to show how critical immune parameters change with therapy and over time could ultimately help transform our understanding of cancer immunobiology. Immune imaging could help make it easier to get the most promising new agents into clinical trials in the most suitable patients and thereby make the regulatory process more rapid, efficient and cost effective.

## Methods

This article contains novel images from several sources. Figure 1 is a deidentified clinical image from the Peter MacCallum Cancer Centre, Melbourne, Australia, that does not require consent for publication, as per institutional policy. It was not acquired as part of a clinical trial. Figure 3 contains images of PET scans of experimental animals that were acquired as part of a research study that was approved by the Ethics Committee of the Peter MacCallum Cancer Centre. Figure 3 also contains deidentified images of PET scans from a clinical trial that was approved by the Ethics Committee of the Peter MacCallum Cancer Centre. For both the animal studies and the human clinical trial, all relevant ethical regulations of the Ethics Committee were complied with. Figure 5 contains deidentified images from a clinical trial conducted at the University of Pennsylvania Medical Center. This study was approved by the hospital Institutional Review Board and all relevant ethical regulations were complied with.
